# Clinical cases of *Cryptosporidium* spp. infections in parrots, canaries and pigeons confirmed by molecular and immunochromatographic methods

**DOI:** 10.2478/jvetres-2026-0008

**Published:** 2026-02-16

**Authors:** Dawid Jańczak, Aleksandra Kornelia Maj, Piotr Górecki, Olga Szaluś-Jordanow, Anna Golke

**Affiliations:** Department of Infectious and Invasive Diseases and Veterinary Administration, Institute of Veterinary Medicine, Faculty of Biological and Veterinary Sciences, Nicolaus Copernicus University, 87-100 Toruń, Poland; Department of Parasitology and Microbiology, Animallab Veterinary Laboratory, 03-430 Warszawa, Poland; Department of Small Animal Diseases with Clinic, Institute of Veterinary Medicine, Warsaw University of Life Sciences-SGGW, 02-776 Warszawa, Poland; Department of Preclinical Sciences, Institute of Veterinary Medicine, Warsaw University of Life Sciences-SGGW, 02-786 Warszawa, Poland

**Keywords:** *Cryptosporidium proventriculi*, diagnostic accuracy, molecular diagnostics, psittacine diseases, zoonotic parasite

## Abstract

**Introduction:**

*Cryptosporidium* spp. is a protozoan parasite capable of infecting all vertebrates worldwide. Infections are typically associated with diarrhoea, although cryptosporidiosis can also involve the respiratory tract. Birds are particularly susceptible to the clinical consequences of *Cryptosporidium* infection, which most commonly include diarrhoea, vomiting, regurgitation, crop inflammation, enterocolitis and poor feather and skin condition.

**Material and Methods:**

In this study, faecal samples from 52 parrots, 3 canaries and 8 fancy pigeons were examined for the presence of *Cryptosporidium* antigen and DNA. One immunochromatographic assay and two amplification methods targeting the 18S rRNA gene fragment were employed (a genus-specific nested PCR and a one-tube nested real-time PCR), and the resulting PCR products were sequenced.

**Results:**

*Cryptosporidium proventriculi* was identified in 15 parrots, while *C. meleagridis* was detected in 1 parrot and 5 pigeons. No mixed infections were reported. The immunochromatographic test sensitivity and specificity were calculated to be 66.7% and 88.1%, respectively.

**Conclusion:**

Given the high prevalence and diversity of *Cryptosporidium* spp. in pet birds, molecular diagnostics are essential for accurate identification and appropriate clinical management. To the best of the authors’ knowledge, this is the first study in Poland aimed at detecting *Cryptosporidium* infections in pet birds.

## Introduction

*Cryptosporidium* is a genus of apicomplexan protozoa infecting a broad spectrum of vertebrate hosts, including fish, amphibians, reptiles, birds and mammals (8). Cryptosporidiosis may progress to a chronic, life-threatening human disease characterised by severe diarrhoea, particularly in children and immunocompromised individuals (19, 20). Approximately 40 species of *Cryptosporidium* have been identified, with *C. hominis* being the predominant cause of human infections; however, zoonotic transmission from animal-associated species such as *C. canis, C. felis, C. suis, C. erinacei, C. parvum, C. meleagridis* and *C. cuniculus* is also well documented (8). Avian cryptosporidiosis was first documented in 1929, when Tyzzer identified oocysts in the caecal epithelium of chickens (46). In contrast to mammals, where the parasites are largely confined to the intestinal tract, avian species frequently harbour infections in the cloacal bursa and respiratory system. In chickens and turkeys, *Cryptosporidium* has been detected in the sinuses, trachea, bronchi, cloaca and bursa of Fabricius (11). The most frequently detected species in poultry are *C. baileyi, C. meleagridis, C. galli* and *C. avium* (8). Among wild birds, six species (*C. andersoni, C. parvum, C. meleagridis, C. avium, C. baileyi* and *C. galli*) and five genotypes (goose I and II and avian I, III and VI) have been documented (48). *Cryptosporidium proventriculi* (formerly avian genotype III) has been reported in Psittaciformes, Passeriformes, toucans and migratory ducks (14, 31, 38). The zoonotic species *C. meleagridis*, a major cause of human cryptosporidiosis, has additionally been identified in wild Anatidae (22) and Columbidae (14, 24). Oocyst shedding is influenced by species, host age and immune status, but typically is in low abundance, which along with the small size of oocysts complicates detection. Conventional diagnosis relies on concentration methods (sucrose flotation or sedimentation) followed by acid-fast staining (Ziehl–Neelsen or Kinyoun) (18). Immunoassays, such as ELISA and rapid immunochromatographic (IC) tests, allow high throughput or rapid screening (4). Molecular approaches, including nested, multiplex and real time PCR as well as PCR and restriction fragment length polymorphism, represent the current gold standard for species identification and genotyping (8, 11, 18, 39, 51). This study aimed to detect *Cryptosporidium* in different species of asymptomatic and clinically affected pet birds by applying two molecular methods in nested PCR form and a rapid immunochromatographic assay.

## Material and Methods

### Sampling

Between March 2024 and April 2025, faecal samples from 63 pet birds comprising 52 parrots, 8 pigeons and 3 canaries were tested in a private veterinary laboratory in Warsaw. There were 22 female birds and 41 males. All samples originated from a single veterinary clinic and were collected during routine visits, which were supplemented by clinical examination and veterinary interview. Each faecal sample was collected individually from each bird and no samples were pooled. The birds were placed in a transport container lined with clean paper towels, and fresh faeces were collected for analysis. All birds included in the study were kept in cages or indoor aviaries without access to the external environment or direct contact with free-living wild birds. None of the pigeons were feral birds.

### Age categorisation of study birds

To facilitate statistical analyses and evaluate age-related trends, the 63 birds included in the study were divided into four age categories. The categorisation was designed to achieve a balance in group size while preserving biologically relevant developmental stages. The following age groups were established: juveniles of ≤1 year (n = 11), early adults of >1 to 2 years (n = 14), mature adults of >2 to 5 years (n = 22) and senior birds >5 years (n = 16). This stratification allowed meaningful statistical comparisons while accounting for developmental and physiological differences associated with age, and differed little from that adopted in previous avian epidemiological and microbiome studies (45).

### Ancillary bacteriological diagnostics

Cloacal, crop, skin and feather-calamus swabs were cultured on blood agar and MacConkey agar (GRASO Biotech, Starogard Gdański, Poland). Plates were incubated aerobically at 37°C for 24–48 h. Bacterial isolates were identified based on colony morphology, Gram staining, biochemical characteristics, and, when necessary, by MALDI-TOF (Microflex LT MALDI-MS Biotyper System; Bruker Daltonics, Bremen, Germany).

### Coproscopy and rapid IC test validation

Parasitological screening was conducted using direct smear and zinc sulphate flotation techniques (7). For the direct smear, ~100 mg of faeces was mixed with 0.9% saline, and a drop was examined microscopically at 100× and 400× magnification. For zinc sulphate flotation, samples were mixed with saturated ZnSO_4_ solution at a specific gravity of 1.31 g/cm^3^, centrifuged for 5 min at 3,000 rpm and examined after 25 min using a coverslip placed on the meniscus. *Cryptosporidium* spp. was detected with a commercial rapid immunoassay (Simple Crypto; Operon, Zaragoza, Spain) per the manufacturer’s instructions. The test demonstrated an estimated sensitivity and specificity of 99.9% relative to microscopic examination. Diagnostic performance was further evaluated against a one-tube nested real-time PCR assay, used as the reference method.

### Isolation of DNA

For molecular analysis, 50–100 mg of fresh faeces was collected and stored frozen. Isolation of DNA was performed using the commercial Genomic Mini AX Stool kit (A&A Biotechnology, Gdańsk, Poland) according to the manufacturer’s protocol. Extracted DNA was resuspended in 200 μL of Tris elution buffer and frozen at –20°C for further tests.

### Nested PCR

A nested PCR was performed according to a previously described protocol (51), using two primer pairs. The first round employed 5′-TTCTAGAGCTAATACATGCG-3′ and 5′-CCCTAATCCTTCGAAACAGGA-3′ primers (expected product: 1325 bp), and the second round used 5′-GGAAGGGTTGTATTTATTAGATAAAG-3′ and 5′-AAGGAGTAAGGAACAACCTCCA-3′ (expected product: ~840 bp) primers. Each 25 μL reaction included StartWarm HS-PCR Mix (A&A Biotechnology), 1.5 μL of each primer (5 μM), 2 μL of template (genomic DNA or first-round product) and nuclease-free water. The PCR conditions were: initial denaturation at 95°C for 3 min; 35 cycles at 95°C for 30 s, 55°C for 45 s and 72°C for 60 s; and a final extension at 72°C for 10 min. The reactions were run on a MultiGene OptiMax thermal cycler (Labnet, Edison, NJ, USA). Genomic DNA of *Cryptosporidium parvum* and ultrapure water served as positive and negative controls, respectively. Nested PCR products were analysed by 2% agarose gel electrophoresis. Amplified products were purified and Sanger sequenced bidirectionally by a commercial provider (Oligo IBB, Warsaw, Poland). Chromatograms were edited using Chromas 2.6.6 (Technelysium, South Brisbane, QLD, Australia) and MEGA 7 (21). Consensus sequences assembled from forward and reverse reads were compared to NCBI GenBank references using BLAST.

### One-tube nested real-time PCR with high-resolution melting

The assay used two primer pairs: the first, described by Johnson *et al*. (17), amplified a ~428 bp fragment of the *Cryptosporidium* 18S ribosomal RNA (rRNA) gene and the second, by Santana *et al*. (39), validated a one-tube nested real-time PCR protocol for avian faecal samples. The reaction was performed in a 20 μL total volume, per the original protocol (39), using 10 μL of EvaGreen RT PCR Mix (A&A Biotechnology), 0.5 μL of each of the NRT18SF (5′-GTTGTTGCAGTTAAAAAGCTCGTAGTTGGATT-3′) and NRT18SR (5′-ACTTTGATTTCTCATAAGGTGCTGAAGG-AGT-3′) primers at 2.5 μM and CPB-DIAGF (5′-AAGCTCGTAGTTGGATTTCTG-3′) and CPB-DIAGR (5′-TAAGGTGCTGAA-GGAGTAAGG-3′) primers at 5 μM, 2 μL of DNA and 6 μL of ultrapure water. Reactions were run on a Gentier 48E Real-Time PCR System (TianLong Biotechnology, Xi’An, China), with fluorescence monitored during the nested PCR stage. The thermal cycling comprised a first step at 95°C for 5 min and 20 cycles of reaction at 95°C for 15 s and 70°C for 30 s, and a second step of 35 cycles of reaction of 95°C for 15 s, 62°C for 30 s and 72°C for 20 s. High-resolution melting curve analysis followed, working with data from 60°C to 95°C at 0.5°C increments and 5-s steps. The decrease in fluorescence intensity with increasing temperature reflected the denaturation of PCR amplicons, and the distinct melting profiles differentiated species based on their melting temperatures. This method enabled discrimination of closely related *Cryptosporidium* species based on differences in DNA sequence composition. The positive control was the DNA of *C. parvum* and the negative control was ultrapure water.

### Statistical analysis

Descriptive statistics were used to summarise the prevalence of *Cryptosporidium* infection among the examined birds. To assess the association between sex (male *vs* female), age and infection status (positive *vs* negative; *Cryptosporidium* species *vs* host species), as well as the relationship between infection, clinical signs and co-infections, two statistical tests were applied. A chi-squared (χ^2^) test of independence was used for a 2 × 2 contingency table, and Fisher’s exact test was performed to confirm the reliability of the results owing to the relatively small sample size. A P-value < 0.05 was considered statistically significant. All statistical analyses were performed using Statistica 13.1 software (Dell, Round Rock, TX, USA).

### Validation of the rapid immunochromatographic assay for detecting *Cryptosporidium* spp. antigen

The validation of the rapid immunochromatographic test was conducted using the one-tube nested real-time PCR as the reference method. This allowed the classification of results as true positives (TP), false positives (FP), true negatives TN) and false negatives (FN). Based on these values, the sensitivity and specificity of the rapid test were calculated using the following formulae: sensitivity = TP / (TP + FN) and specificity = TN / (TN + FP).

## Results

### Coproscopy and *Cryptosporidium* spp. detection

**Fig. 1.**
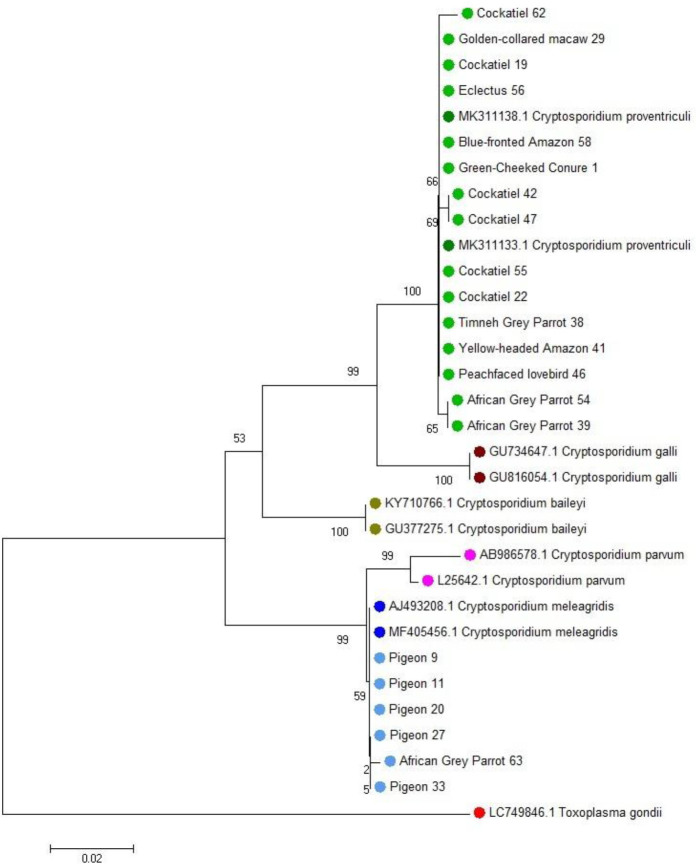
Phylogenetic relationships of 32 nucleotide sequences of *Cryptosporidium* species isolated from pet birds. The analysis was performed using the neighbour-joining method based on nucleotide sequences of the small-subunit ribosomal RNA gene. The evolutionary distances were computed using the Kimura two-parameter method and are the numbers of base substitutions per site. Two-letter and six-digit codes are GenBank accession numbers. *Toxoplasma gondii* was used as the outgroup

**Fig. 2.**
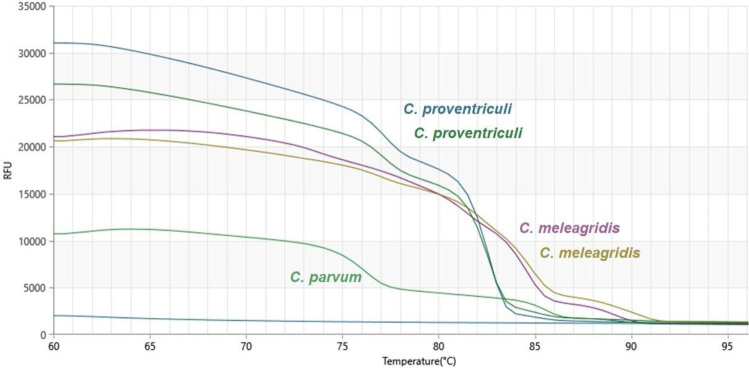
Melting curves obtained during qPCR-high-resolution melting analysis of the products of amplification of isolates of *Cryptosporidium* spp. from the faeces of pet birds. The paired curves for *C. meleagridis* and *C. proventriculi* indicate technical replicates or distinct isolates. RFU – relative fluorescence units

**Fig. 3.**
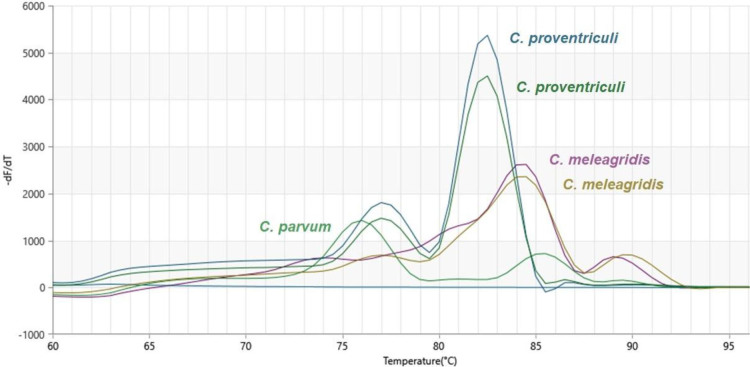
Derivative melting curve analysis of the products of amplification of isolates of *Cryptosporidium* spp from the faeces of pet birds. The paired curves for *C. meleagridis* and *C. proventriculi* indicate technical replicates or distinct isolates. –dF/dT – negative first derivative of fluorescence with respect to temperature

Microscopic examination revealed the presence of protozoa in two pigeons: *Eimeria* spp. oocysts in one individual and *Trichomonas* spp. trophozoites in the other. No helminth eggs were detected in any of the examined samples. Using the one-tube nested real-time PCR assay, *Cryptosporidium* spp. DNA was detected in 33.3% of the faecal samples (21/63), which were those from 16 parrots and 5 pigeons. The nested PCR method identified *Cryptosporidium* spp. in 25.4% of samples (16/63), which were those from 13 parrots and 3 pigeons. In comparison, the antigen detection test yielded positive results in 30.2% of cases (19/63). None of the faecal samples from canaries tested positive in the applied methods. Additionally, co-infections were observed in two pigeons: one with *Eimeria* spp. and *Cryptosporidium* spp., and the other with *Trichomonas* spp. and *Cryptosporidium* spp. Sequencing of the products obtained from both the nested PCR and the one-tube nested real-time PCR enabled the identification of the parasite species. *Cryptosporidium proventriculi* was detected in 15 samples from parrots, while *C. meleagridis* was identified in 5 samples from pigeons and 1 from a parrot (Figs 1–3). The complete dataset is available in Supplementary Table S1.

### The relationship between *Cryptosporidium* spp. infection, bacterial co-infections and presence of clinical symptoms

*Cryptosporidium* spp. was detected in 21 of the 63 birds (33.3%), all of which exhibited various clinical symptoms. Among the remaining 42 birds, 28 showed clinical signs despite testing negative for *Cryptosporidium* spp., while 14 birds had no symptoms and tested negative. The chi-squared test revealed a statistically significant association (χ2 = 7.17, P-value = 0.0074). Moreover, Fisher’s exact test confirmed an important relationship (P-value = 0.0026), indicating that *Cryptosporidium*-infected birds were significantly more likely to present with clinical signs than uninfected individuals. These findings support the hypothesis that *Cryptosporidium* infection was significantly associated with clinical symptoms in the examined birds (P-value < 0.01). Fisher’s exact test also revealed a statistically significant association between *Cryptosporidium* infection and concurrent bacterial or other protozoan infections (P-value = 0.0002, odds ratio = 9.17), suggesting that co-infections were substantially more likely in *Cryptosporidium*-positive birds. Although clinical signs were observed in most birds with bacterial infections (either alone or in co-infection), chi-squared analysis did not reveal a statistically significant association between infection type and clinical symptom occurrence (χ2 = 3.12, P-value = 0.21).

Diarrhoea, poor skin condition, vomiting and/or regurgitation and sneezing and/or upper respiratory tract disorders were selected from the most frequently observed clinical signs for statistical analysis in relation to *Cryptosporidium* infection. In the cohort of 63 pet birds examined, diarrhoea was observed in 6 individuals, of which 4 tested positive for *Cryptosporidium*. Among the 57 birds without diarrhoea, 15 were positive for the parasite. Statistical analysis using the chi-squared test revealed a significant association between diarrhoea and *Cryptosporidium* infection (χ^2^ = 4.20, degrees of freedom (df) = 1, P-value < 0.05), suggesting that the presence of *Cryptosporidium* is significantly associated with the occurrence of diarrhoea in the studied population. This finding supports the hypothesis that *Cryptosporidium* infection may have contributed to gastrointestinal disturbances in these birds. Skin in poor condition was identified in 5 birds, all of which tested positive for *Cryptosporidium*, whereas 16 out of 58 birds with healthy skin were infected. The association between poor skin condition and *Cryptosporidium* infection was highly significant (χ2 = 10.84, df = 1, P-value < 0.01), indicating a potential link between the parasite and dermatological manifestations. This may reflect systemic effects of the infection or compromised health status in affected individuals. Conversely, only 2 of the 8 birds noted with vomiting and/or regurgitation tested positive for *Cryptosporidium*, while 19 of the 55 birds without these clinical signs were infected. The chi-squared test did not demonstrate a statistically significant relationship between vomiting/regurgitation and *Cryptosporidium* infection (χ2 = 0.28, df = 1, P-value > 0.05), suggesting that these symptoms were unlikely to be directly associated with the parasite in this cohort. Similarly, birds exhibiting sneezing and/or upper respiratory tract disorders showed an identical non-significant result (χ2 = 0.28, df = 1, P-value > 0.05), indicating no significant association between respiratory symptoms and *Cryptosporidium* infection. These findings imply that while *Cryptosporidium* infection may have been linked to certain clinical signs such as diarrhoea and dermatological changes, other symptoms observed in the examined birds were likely attributable to different aetiologies or multifactorial causes.

### The relationship between *Cryptosporidium* spp. infection and bird sex

Infection with *Cryptosporidium* spp. was detected in 9 of the 22 female birds (40.9%) and 12 of the 41 males (29.3%). Statistical analysis was conducted to assess the association between sex and infection status. A chi-squared test of independence revealed no statistically significant relationship between sex and *Cryptosporidium* infection (χ2 = 0.87, df = 1, P-value = 0.35). Fisher’s exact test also confirmed the lack of significant association (P-value = 0.41). These results indicate that, within this sample, sex was not an important factor influencing the prevalence of *Cryptosporidium* infection.

### The relationship between *Cryptosporidium* spp. infection and age

The prevalence of *Cryptosporidium* infection in the youngest group was 45.5%, in the early adult group was 28.6%, in the mature adult group was 31.8% and in the senior group was 31.3%. A chi-squared test of independence showed no statistically significant association between age group and *Cryptosporidium* infection (χ2 = 0.92, df = 3, P-value = 0.82), indicating that age was not an important factor influencing the likelihood of infection in this population.

### Association of *C. proventriculi* infection with bird taxonomic groups

In this study, *C. proventriculi* was detected exclusively in parrots (Psittacidae family, including *Psittacus, Nymphicus* and other genera), with 15 out of 52 parrots (30.8%) but none of the 8 pigeons (Columbidae) testing positive. A Fisher’s exact test was performed to evaluate whether the occurrence of *C. proventriculi* differed significantly between the two families. The test showed a statistically significant association (P-value = 0.048), indicating that *C. proventriculi* was significantly more frequent in parrots than pigeons. Within the Psittacidae family itself, *C. proventriculi* was detected in 6 out of 20 cockatiels (*Nymphicus hollandicus*; 30.0%) and 9 out of 43 individuals of other genera (20.9%), although Fisher’s exact test did not reveal a statistically significant difference between these groups (P-value = 0.327). These results suggest that although *C. proventriculi* was most frequently identified in cockatiels, the difference in prevalence was not statistically significant within the studied population (Table 2).

**Table 2.** Results of the PCR test for the presence of two *Cryptosporidium* spp. in the studied bird group

Species	n	*C. proventriculi*		*C. meleagridis*	
		n (+)	%	n (+)	%
African grey parrot (*Psittacus erithacus*)	3	2	66.7	1	33.3
Blue-and-yellow macaw (*Ara ararauna*)	2	0	0.0	0	0.0
Blue-fronted amazon (*Amazona aestiva*)	2	1	50.0	0	0.0
Blue-headed pionus (*Pionus menstruus*)	1	0	0.0	0	0.0
Budgerigar (*Melopsittacus undulatus*)	4	0	0.0	0	0.0
Canary (*Serinus canaria*)	3	0	0.0	0	0.0
Cockatiel (*Nymphicus hollandicus*)	20	6	30	0	0.0
Eastern rosella (*Platycercus eximius*)	2	0	0.0	0	0.0
Moluccan eclectus (*Eclectus roratus*)	1	1	100	0	0.0
Golden-collared macaw (*Primolius auricollis*)	1	1	100	0	0.0
Green-cheeked conure (*Pyrrhura molinae*)	3	1	33.3	0	0.0
Peach-faced lovebird (*Agapornis roseicollis*)	4	1	25	0	0.0
Pigeon (*Columba livia*)	8	0	0.0	5	62.5
Plum-headed parakeet (*Psittacula cyanocephala*)	2	0	0.0	0	0.0
Rainbow Lorikeet (*Trichoglossus moluccanus*)	2	0	0.0	0	0.0
Ring-necked parakeet (*Psittacula krameri*)	1	0	0.0	0	0.0
Senegal parrot (*Poicephalus senegalus*)	1	0	0.0	0	0.0
Sun conure (*Aratinga solstitialis*)	1	0	0.0	0	0.0
Timneh grey parrot (*Psittacus erithacus timneh*)	1	1	100	0	0.0
Yellow-headed amazon (*Amazona oratrix*)	1	1	100	0	0.0
Total	63	15	23.8	6	9.52

### Validation of the rapid immunochromatographic assay for detecting *Cryptosporidium* spp. antigens

The nested real-time PCR identified 21 positive and 42 negative individuals. The results were divided into groups (Table 3), and the assay’s sensitivity and specificity were 66.7% and 88.1%, respectively.

**Table 3.** Diagnostic performance of the immunochromatographic assay evaluated against one-tube nested real-time PCR for detection of *Cryptosporidium* spp. in avian faeces

	Positive (ref. method n = 21)	Negative (ref. method n = 42)
IC rapid assay positive	14	5
IC rapid assay negative	7	37

## Discussion

Infections caused by *Cryptosporidium* spp. have been reported in numerous bird species in the Passeriformes, Psittaciformes and Columbiformes orders worldwide (31, 32, 33, 34, 38). *Cryptosporidium proventriculi*, previously referred to as avian genotype III, appears to be the most prevalent species infecting parrots in both the New and Old Worlds (9, 14, 16, 23, 25, 26, 27, 29, 30, 33, 34, 35, 37, 38). In our study, *C. proventriculi* was detected exclusively in faecal samples from parrots. This parasite species is a known cause of proventricular inflammation and chronic vomiting in some psittacine birds. Makino *et al*. (25) confirmed *C. proventriculi* infection in 13 of 37 peach-faced lovebirds using PCR, noting chronic vomiting, weight loss and radiographic signs such as isthmus enlargement, narrowed lumens and thickened proventricular walls. In our study, infected parrots presented with vomiting, regurgitation, obesity, diarrhoea, beak overgrowth and feather degradation. Dermatitis and feather plucking were noted, with one bird having a wing wound. Laboratory tests indicated bacterial infections of the skin, intestines and crop. In some cases, ultrasound and blood analysis confirmed hepatic steatosis, cirrhosis and hepatitis. Ravich *et al*. (35) performed a histopathologic analysis of 34 psittacine birds – 12 cockatiels, 18 lovebirds (2 *Agapornis roseicollis* and 16 of unknown species) and four parrotlets (*Forpus* sp.). Although histopathological examinations of birds that had died suddenly revealed the presence of *Cryptosporidium* and associated mucosal hyperplasia of the proventriculus, other lesions were also observed, suggesting that the *Cryptosporidium* infection was secondary and should not have been considered the primary cause of death. However, the researchers emphasised that in 11 birds, the lesions were exclusively associated with gastrointestinal cryptosporidiosis. *Cryptosporidium galli* and *C. proventriculi* are considered two of the most pathogenic *Cryptosporidium* species in birds and cause clinical signs related to the gastrointestinal tract such as vomiting, diarrhoea and weight loss (11, 28, 33, 37, 41). In our study, a statistically significant association between diarrhoea and *C. proventriculi* infection was observed, consistently with the findings of Pangeossi *et al*. (33), who reported a similar association in a group of 100 cockatiels (P-value = 0.017). The same authors also identified a significant relationship between *Cryptosporidium* infection and the birds’ origin, noting that owned birds had a higher prevalence of infection than those from pet shops. In our study, all examined birds were privately owned.

*Cryptosporidium galli* has been identified in poultry and birds of the Passeriformes order (29, 30, 39). Studies conducted by da Silva *et al*. (41) indicate that *C. galli* is commonly found in great-billed seed finches (*Oryzoborus maximiliani*), lesser seed finches (*Oryzoborus angolensis*), ultramarine grosbeaks (*Cyanocompsa brissonii*) and rusty-collared seedeaters (*Sporophila collaris*). However, its virulence appears to be low. Pathogenic effects were observed only in one aviary, where bird mortality was associated with coinfection with *E. coli*. In our study, no sample was *C. galli* positive. Although no cases of mortality were observed among our patients, the vast majority of birds infected with *Cryptosporidium* presented with concurrent bacterial infections of the skin, intestines or crop, which may be considered co-infections with associated clinical manifestations. Clinical signs were frequently observed in the study birds with bacterial infections, whether present alone or in combination with other pathogens; however, statistical analysis did not show a significant association between the type of infection and the occurrence of clinical symptoms.

*Cryptosporidium meleagridis* was first described in young turkeys (43), but has also been found in chickens, ducks, pet birds and wild birds (2, 5, 12, 13, 23, 26, 34, 48). In our study, it was detected in five pigeons and one African grey parrot. Reports on *Cryptosporidium* genotypes in pigeons are limited (24, 32, 41) and correspondingly, those focusing on pigeons in Poland are sparse (13). As synanthropic birds common in urban and rural areas, pigeons may play a key role in pathogen transmission. In Chile, Briceño *et al*. (5) reported *C. meleagridis* in invasive monk parakeets from Santiago, and their finding points to the need for ongoing surveillance in widely distributed synanthropic species. *Cryptosporidium meleagridis* is among the three species in the genus most frequently linked to human cryptosporidiosis (8, 42). In our study, it was found in an African grey parrot with chronic respiratory infection caused by *Mycobacterium* sp. Similarly, Huh *et al*. (15) reported chronic diarrhoea in an AIDS patient coinfected with *Mycobacterium ulcerans* and *Cryptosporidium* spp. In Poland, human cryptosporidiosis caused by *C. meleagridis* was reported in four patients: two immunocompromised children (3, 50), an adult HIV-positive woman suffering from chronic diarrhoea (49) and an immunocompetent patient with colon adenocarcinoma (19). All these cases highlight the importance of comprehensive diagnostic workups, particularly in immunocompromised patients suffering from chronic respiratory infections or persistent diarrhoea.

Detection of *Cryptosporidium* spp. by PCR offers greater sensitivity than traditional microscopy or immunoassays, enables high-throughput analysis and identifies the parasite precisely to species level (6, 27, 39, 47, 51). Key genetic targets for species differentiation include small-subunit rRNA, Cryptosporidium oocyst-wall protein, 70-kDa heatshock protein, thrombospondin-related anonymous protein C2 and the actin gene (14, 30, 36, 51). The most widely used method for diagnosing cryptosporidiosis and conducting phylogenetic analysis is amplification of the 18S rRNA gene fragment (13, 26, 29, 37, 51). Our study utilised two PCR-based approaches which amplified this fragment: nested PCR (51) and one-tube nested real-time PCR (17, 39). However, for initial screening for *Cryptosporidium* spp., we applied a rapid immunochromatographic test targeting protozoan antigens. Antigen presence was detected in 30.2% (19/63) of faecal samples. Using molecular methods, *Cryptosporidium* spp. DNA was identified in 25.4% (16/63) of samples with nested PCR and in 33.3% (21/63) in the one-tube nested real-time PCR. As all faecal samples were tested using the same diagnostic methods, we validated the rapid test against the one-tube nested real-time PCR, the chain reaction being considered the reference based on our findings and those of Santana *et al*. (39). The rapid immunochromatographic test showed a sensitivity of 66.7% and specificity of 88.1%, indicating moderate sensitivity and high specificity for detecting *Cryptosporidium* antigen in avian faeces. Although the test was designed for human samples, similar products from the same manufacturer have been used in dogs and cats and have achieved sensitivity of 46.1% and specificity of 99.0% (4). Abdou *et al*. (1) also used a rapid immunochromatographic test in cattle and detected *Cryptosporidium* in 23% (92/400) of samples, establishing the test’s sensitivity at 74.07%. These tests have nevertheless been associated with high false-positive rates. In our study, five samples tested positive for antigen in the rapid IC assay but negative in both PCR tests. Conversely, seven PCR-positive samples lacked detectable antigen in rapid IC. These discrepancies and the modest test performance in birds may result from interference by substances in avian faeces, such as uric acid, which is absent from human stool. The current literature offers limited data on rapid immunochromatographic test performance in birds. One study assessing occult blood tests in cockatiels suggested that false positives could stem from diet, peroxidase activity or minor gastrointestinal bleeding (10). These findings indicate that there is a need for reliable diagnostic tools for *Cryptosporidium* spp. detection in avian veterinary practice, particularly since no effective therapy is available for avian cryptosporidiosis. Early detection enables timely isolation and biosecurity measures to prevent spread. Therapy with paromomycin has demonstrated partial efficacy in chickens, reducing oocyst shedding, although it may predispose birds to secondary fungal infections (44). Consequently, control strategies rely primarily on prophylaxis, afforded jointly through strict biosecurity and hygiene to negate the advantage provided by the marked environmental resistance of oocysts, and through immune support to at-risk birds (40).

## Conclusion

*Cryptosporidium* spp. infections are widespread in pet and wild birds, with clinical involvement often extending beyond the gastrointestinal tract and frequently complicated by bacterial co-infections. Among psittacines, *C. proventriculi* was the dominant species, while *C. meleagridis*, a zoonotic pathogen of major public health concern, was also identified in pigeons and parrots. Given the absence of effective therapies and the risk of secondary fungal infections associated with paromomycin, preventive measures like biosecurity, hygiene, nutritional balance and immune support remain essential. Importantly, zoonotic species such as *C. meleagridis* warrant molecular confirmation in birds, particularly those owned by immunocompromised individuals. Detection of *Cryptosporidium* in avian patients should prompt comprehensive clinical evaluation, as infection may signal underlying systemic disease.

## Supplementary Material

Supplementary Material Details
