# Supplementary material for: Clinical cases of Cryptosporidium spp. infections in parrots, canaries and pigeons confirmed by molecular and immunochromatographic methods
Source: J Vet Res. 2026 Feb 16;70(1):91–100. doi: 10.2478/jvetres-2026-0008 (PMC13054763; doi:10.2478/jvetres-2026-0008)
Supplement: Supplementary file 1 — Supplementary Material Details [file jvetres-2026-0008_sm.pdf]

Supplementary Table S1. Age, sex, clinical signs, diagnoses, additional test results and findings of *Cryptosporidium* examination in the studied birds

	Species	Age	Sex	Symptoms noticed by the owner	Diagnosis / additional clinical findings	Microscopy	IC assay	nested PCR	One-tube nested real-time PCR
1	Green-cheeked conure (*Pyrrhura molinae*)	2Y	M	cracked beak, beak overgrowth, malocclusion	flaky beak, hepatic steatosis; choanal swab culture: *Streptococcus bovis*	−	+	*C. p.*	*C. p.*
2	Cockatiel (*Nymphicus hollandicus*)	6M	F	-	hepatomegaly	−	−	-	-
3	Cockatiel (*Nymphicus hollandicus*)	1Y	F	-	crop swab culture: *Staphylococcus haemolyticus*	−	−	-	-
4	Budgerigar (*Melopsittacus undulatus*)	1Y	F	-	-	−	−	-	-
5	Budgerigar (*Melopsittacus undulatus*)	2Y	M	dyspnoea, watery eye for 3 months	dyspnoea, conjunctivitis, intensified upper respiratory tract sounds	−	−	-	-
6	Canary (*Serinus canaria*)	5Y	M	feather cysts	recurrent feather cysts and bacterial dermatitis	−	−	-	-
7	Blue-and-yellow macaw (*Ara ararauna*)	6Y	F	beak rubbing, regurgitation	recurrent bacterial sinusitis and crop inflammation; cloacal swab culture: *Enterobacter cloacae* spp. *cloacae*	−	−	-	-
8	Canary (*Serinus canaria*)	2Y	F	lethargy, fluffed feathers, feather plucking around the neck area	bacterial crop inflammation; cloacal swab culture: methicillin-resistant *Staphylococcus epidermidis*	−	−	-	-
9	Pigeon (*Columba livia*)	1Y	M	anorexia	loose stool; faecal culture: *Enterococcus columbae* and *Staphylococcus saprophyticus*	−	+	-	*C. m.*
10	Pigeon (*Columba livia*)	3Y	F	-	-	−	+	-	-
11	Pigeon (*Columba livia*)	2Y	M	ocular discharge	conjunctivitis, intestinal coccidiosis due to *Eimeria* spp.	+	+	*C. m.*	*C. m.*
12	Canary (*Serinus canaria*)	3Y	M	-	-	−	−	-	-
13	Eastern rosella (*Platycercus eximius*)	2Y	M	-	-	−	−	-	-
14	Eastern rosella (*Platycercus eximius*)	3Y	M	-	-	−	−	-	-
15	Cockatiel (*Nymphicus hollandicus*)	2Y	M	-	crop swab culture: *Staphylococcus saprophyticus*	−	−	-	-
16	Cockatiel (*Nymphicus hollandicus*)	5Y	M	-	-	−	−	-	-
17	Plum-headed parakeet (*Psittacula cyanocephala*)	3Y	F	-	-	−	−	-	-
18	Plum-headed parakeet (*Psittacula cyanocephala*)	3Y	F	-	-	−	−	-	-
19	Cockatiel (*Nymphicus hollandicus*)	3Y	M	recurrent diarrhoea and regurgitation	poor skin condition, hepatitis, trichomoniasis, *Trichomonas* spp.	+	+	*C. p.*	*C. p.*
20	Pigeon (*Columba livia*)	2Y	M	poor skin condition	hepatitis, recurrent skin abscesses, brittle feathers; feather-calamus culture – macrolide-, lincosamide- and streptogramin B-resistant *Staphylococcus aureus*	−	+	*C. m.*	*C. m.*
21	Pigeon (*Columba livia*)	2Y	M	beak overgrowth, anorexia	hepatic steatosis, traumatic beak overgrowth	−	+	-	-

22	Cockatiel (*Nymphicus hollandicus*)	1Y	M	vomiting, diarrhoea	bacterial crop inflammation and enterocolitis; cloacal swab culture: methicillin-susceptible *Staphylococcus aureus*	−	+	*C. p.*	*C. p.*
23	Peach-faced love-bird (*Agapornis roseicollis*)	4Y	M	loss of balance, inability to fly, seizure-like symptoms	neurological signs; crop swab culture: *Klebsiella pneumoniae*; cloacal swab culture: *Enterococcus casseliflavus*	−	+	-	-
24	Blue-fronted amazon (*Amazona aestiva*)	7Y	M	aggression, unable to fly	hepatitis with steatosis; cloacal swab culture: no bacteria growth	−	−	-	-
25	Blue-and-yellow macaw (*Ara ararauna*)	6Y	F	aggression, anorexia, large faecal masses	bacterial enterocolitis; cloacal swab culture: *Staphylococcus saprophyticus* ssp. *saprophyticus* and *Staphylococcus salivarius*	−	−	-	-
26	Cockatiel (*Nymphicus hollandicus*)	5Y	M	reduced appetite, occasional regurgitation	hepatic steatosis, liver cirrhosis	−	−	-	-
27	Pigeon (*Columba livia*)	3M	M	upper respiratory signs, diarrhoea	intensified respiratory sounds, nasal discharge, loose stool; beak fissure and nasal swab: *Chlamydophila psittaci* (detected by real-time PCR); choanal swab culture: *Staphylococcus petrasii*	−	+	-	*C. m.*
28	Blue-headed pionus (*Pionus menstruus*)	6M	M	poor feather condition	zinc intoxication; cloacal swab culture: no bacteria growth	−	−	-	-
29	Golden-collared macaw (*Primolius auricollis*)	6Y	M	poor feather condition	hepatitis and enterocolitis; choanal swab culture: *Citrobacter freundii*	−	−	*C. p.*	*C. p.*
30	Cockatiel (*Nymphicus hollandicus*)	9Y	F	obesity, feather yellowing	liver cirrhosis and hepatitis; cloacal swab culture: *Enterococcus columbae*	−	−	-	-
31	Cockatiel (*Nymphicus hollandicus*)	16 Y	M	beak overgrowth, poor feather condition	hepatitis; choanal swab culture: *Klebsiella pneumoniae*	−	−	-	-
32	Cockatiel (*Nymphicus hollandicus*)	20 Y	M	enlarged abdomen	hepatomegaly, hepatitis; cloacal swab culture: *Enterococcus casseliflavus*	−	−	-	-
33	Pigeon (*Columba livia*)	5M	M	anorexia	bacterial crop inflammation and enterocolitis; cloacal swab culture: β-haemolytic *E. coli*	−	+	*C. m.*	*C. m.*
34	Green-cheeked conure (*Pyrrhura molinae*)	2Y	F	-	recurrent hepatitis	−	−	-	-
35	Green-cheeked conure (*Pyrrhura molinae*)	3Y	M	sneezing	recurrent bacterial crop inflammation; choanal swab culture: *Enterobacter cloacae*	−	−	-	-
36	Ring-necked parakeet (*Psittacula krameri*)	9Y	F	sneezing, discharge from right nostril	recurrent sinusitis and nasal discharge	−	−	-	-
37	Cockatiel (*Nymphicus hollandicus*)	8Y	F	diarrhoea	bacterial enterocolitis	−	−	-	-
38	Timneh grey parrot (*Psittacus erithacus timneh*)	30 Y	F	feather plucking, wound under left wing	necrotising inflammation of the patagial membrane; feather-calamus culture: no bacteria growth	−	−	-	*C. p.*
39	African grey parrot (*Psittacus erithacus*)	5Y	F	wound on the rump, feather plucking	necrotising dermatitis; wound swab culture: *Enterococcus faecalis*, cloacal swab culture: *Enterococcus faecalis*	−	+	*C. p.*	*C. p.*
40	Pigeon (*Columba livia*)	2Y	M	large, loose faeces	bacterial enterocolitis	−	−	-	-
41	Yellow-headed amazon (*Amazona oratrix*)	4Y	F	poor feather and beak condition, obesity	liver cirrhosis, beak overgrowth	−	−	*C. p.*	*C. p.*
42	Cockatiel (*Nymphicus hollandicus*)	2Y	M	lethargy, occasional ocular discharge, sneezing	hepatic steatosis, sinusitis	−	−	*C. p.*	*C. p.*

43	Cockatiel (*Nymphicus hollandicus*)	2Y	M	occasional sneezing	sinusitis	−	−	-	-
44	Budgerigar (*Melopsittacus undulatus*)	3Y	M	paresis, weight loss	osteomyelitis due to *Enterococcus* spp. infection	−	−	-	-
45	Budgerigar (*Melopsittacus undulatus*)	3Y	M	-		−	−	-	-
46	Peach-faced love-bird (*Agapornis roseicollis*)	6Y	M	beak overgrowth	hepatitis, fungal infection of the beak; *Cladosporium* spp. growth	−	−	*C. p.*	*C. p.*
47	Cockatiel (*Nymphicus hollandicus*)	3Y	M	recurrent diarrhoea and regurgitation	hepatitis	−	+	*C. p.*	*C. p.*
48	Sun conure (*Aratinga solstitialis*)	6Y	M	feather plucking, self-mutilation, aggression	liver cirrhosis and hepatitis; cloacal swab culture: no bacteria growth	−	−	-	-
49	Senegal parrot (*Poicephalus senegalus*)	9Y	M	recurrent diarrhoea	bacterial enterocolitis, liver cirrhosis; cloacal swab culture: *Enterococcus durans*	−	+	-	-
50	Cockatiel (*Nymphicus hollandicus*)	2Y	M	recurrent regurgitation, crop gas distension	bacterial crop inflammation	−	−	-	-
51	Cockatiel (*Nymphicus hollandicus*)	2Y	M	recurrent regurgitation, vomiting	bacterial crop inflammation	−	−	-	-
52	Peach-faced love-bird (*Agapornis roseicollis*)	6Y	M	feather plucking	ulcerative dermatitis, hypothyroidism	−	−	-	-
53	Peach-faced love-bird (*Agapornis roseicollis*)	3Y	M	recurrent regurgitation	bacterial crop inflammation; crop swab culture: β-haemolytic *E. coli*; choanal swab culture: *Staphylococcus auricularis*, *Enterococcus columbae*	−	−	-	-
54	African grey parrot (*Psittacus erithacus*)	3Y	F	wing feather plucking, inability to fly	ulcerative dermatitis; skin swab culture: no bacteria growth	−	−	-	*C. p.*
55	Cockatiel (*Nymphicus hollandicus*)	9Y	F	feather plucking	bacterial dermatitis, tibiotarsal arthritis, liver cirrhosis; crop swab culture: *Pseudomonas aeruginosa*, *Pseudomonas monteilii*, *Acineto-bacter pitti*, cloacal swab culture: *Corynebacterium falsenii*	−	−	-	*C. p.*
56	Moluccan eclectus (*Eclectus roratus*)	1Y	M	poor feather and beak condition, obesity	hepatic steatosis, bacterial enterocolitis; cloacal swab culture: *Candida albicans*, choanal swab culture: *Acinetobacter baumannii*	−	+	*C. p.*	*C. p.*
57	Cockatiel (*Nymphicus hollandicus*)	1Y	M	poor feather condition	bacterial enterocolitis	−	−	-	-
58	Blue-fronted amazon (*Amazona aestiva*)	10M	F	poor feather and beak condition, obesity, loose faeces	hepatic steatosis, bacterial enterocolitis; crop swab culture: *Streptococcus suis*, cloacal swab culture: *Bacillus cereus*	−	+	*C. p.*	*C. p.*
59	Rainbow lorikeet (*Trichoglossus moluccanus*)	3Y	M	poor feather condition	cloacal swab culture: no bacteria growth	−	−	-	-
60	Rainbow lorikeet (*Trichoglossus moluccanus*)	3Y	M	poor feather condition	cloacal swab culture: no bacteria growth	−	−	-	-
61	Cockatiel (*Nymphicus hollandicus*)	5Y	F	loose faeces	liver cirrhosis	−	+	-	-
62	Cockatiel (*Nymphicus hollandicus*)	4Y	F	loose faeces	liver cirrhosis; cloacal swab culture: no bacteria growth	−	+	*C. p.*	*C. p.*

63	African grey parrot (*Psittacus erithacus*)	15 Y	F	unable to fly for years, recent itching of back	dry skin, liver cirrhosis, respiratory tract infection due to *Mycobacterium terrae* (Löwenstein–Jensen medium culture; rpoB – identification by sequencing); cloacal swab culture: *Staphylococcus epidermidis*; choanal swab: *Streptococcus* spp.	–	+	*C. m.*	*C. m.*

IC – immunochromatographic; *C. p.* – *Cryptosporidium proventriculi*; *C. m.* – *Cryptosporidium meleagridis*
